# Efficiency and safety gains from bedside electronic transfusion checks in four hospitals in England

**DOI:** 10.1111/bjh.70681

**Published:** 2026-08-06

**Authors:** Nathan Proudlove, Florence Oyekan, Montasir Ahmed, Louise Bowles, Yan Feng, Helinor McAleese, Josephine McCullagh, Michael F. Murphy, Simon J. Stanworth, Laura Green

**Affiliations:** ^1^ Alliance Manchester Business School University of Manchester Manchester UK; ^2^ Barts Health NHS Trust London UK; ^3^ Blizard Institute, Barts and the London School of Medicine and Dentistry Queen Mary University of London London UK; ^4^ Wolfson Institute of Population Health Queen Mary University of London London UK; ^5^ NHS Blood and Transplant London UK; ^6^ Oxford University Hospitals NHS Foundation Trust Oxford UK; ^7^ Blood and Transplant Research Unit, Nuffield Division of Clinical Laboratory Sciences, Radcliffe Department of Medicine University of Oxford Oxford UK

**Keywords:** haematology, manuscript, research

## Abstract

Bedside electronic transfusion check (BETC) systems support safer practice by aiding staff in patient identification during group and screen (G&S) sample labelling and blood component administration, steps where most transfusion errors occur. While the safety rationale is clear, the significant costs necessitate robust cost–benefit evaluation. During phased BETC implementation in four London hospitals, we mapped in detail transfusion workflow, quantifying staff time saved under three modes of technology adoption which enable three corresponding states of organisational use: Mode‐A (baseline: manual‐only); Mode‐B (partial technology‐capability implementation, mixed coverage and staff uptake); and Mode‐C (full implementation, wide coverage and 100% uptake). Benefits depend on implementation mode and uptake. Across the whole transfusion pathway, estimated staff‐time saved per blood unit (including fewer G&S) was 9.3 min (20% reduction) under Mode‐B and 17.3 min (38%) under Mode‐C compared with baseline. Estimated annual time savings across the four hospitals were 8950 h (Mode‐B) and 16 650 h (Mode‐C), including fewer G&S samples (3800 Mode‐B; 26 950 Mode‐C) because of fewer rejections with BETC and the potential to remove the second G&S sample policy under Mode‐C. BETC also substantially improved staff compliance with safety protocols. Overall, BETC can deliver significant efficiency gains alongside safety improvements, supporting the case for wider adoption.

## INTRODUCTION

Blood transfusion is the most common medical procedure in hospitalised patients and errors, often caused by positive patient identification (PPID) during group and screen (G&S) sample collection/labelling or blood administration, can have severe consequences.^1^ The use of bedside electronic transfusion checking (BETC) systems can mitigate these errors by providing digital support to staff for performing PPID during G&S sample labelling and blood administration and record blood use fate (traceability), through barcode scanning of patient wristbands, blood component units and staff ID badges. Scanning is performed with personal digital assistant (PDA) devices connected to a hospital's electronic blood management system (EBMS),^2^, ^3^ enabling full ‘vein‐to‐vein’ digital transfusion support. Without BETC, these steps remain manual, time‐consuming and error‐prone, risking G&S sample rejection and the wrong blood being given to the wrong patient, which can have catastrophic consequences.

Despite national recommendations,^4^, ^5^ the uptake of BETC remains low with only one‐third of hospitals in England using BETC in 2023, compared with 56% adoption for electronic blood fridges.^2^ Reported barriers included a lack of funding and leadership support in hospitals and limited health economic evidence.^2^ Previous evaluations have largely focused on safety outcomes, with little robust data on resource use, workflow or cost‐effectiveness.^3^

**FIGURE 1:**
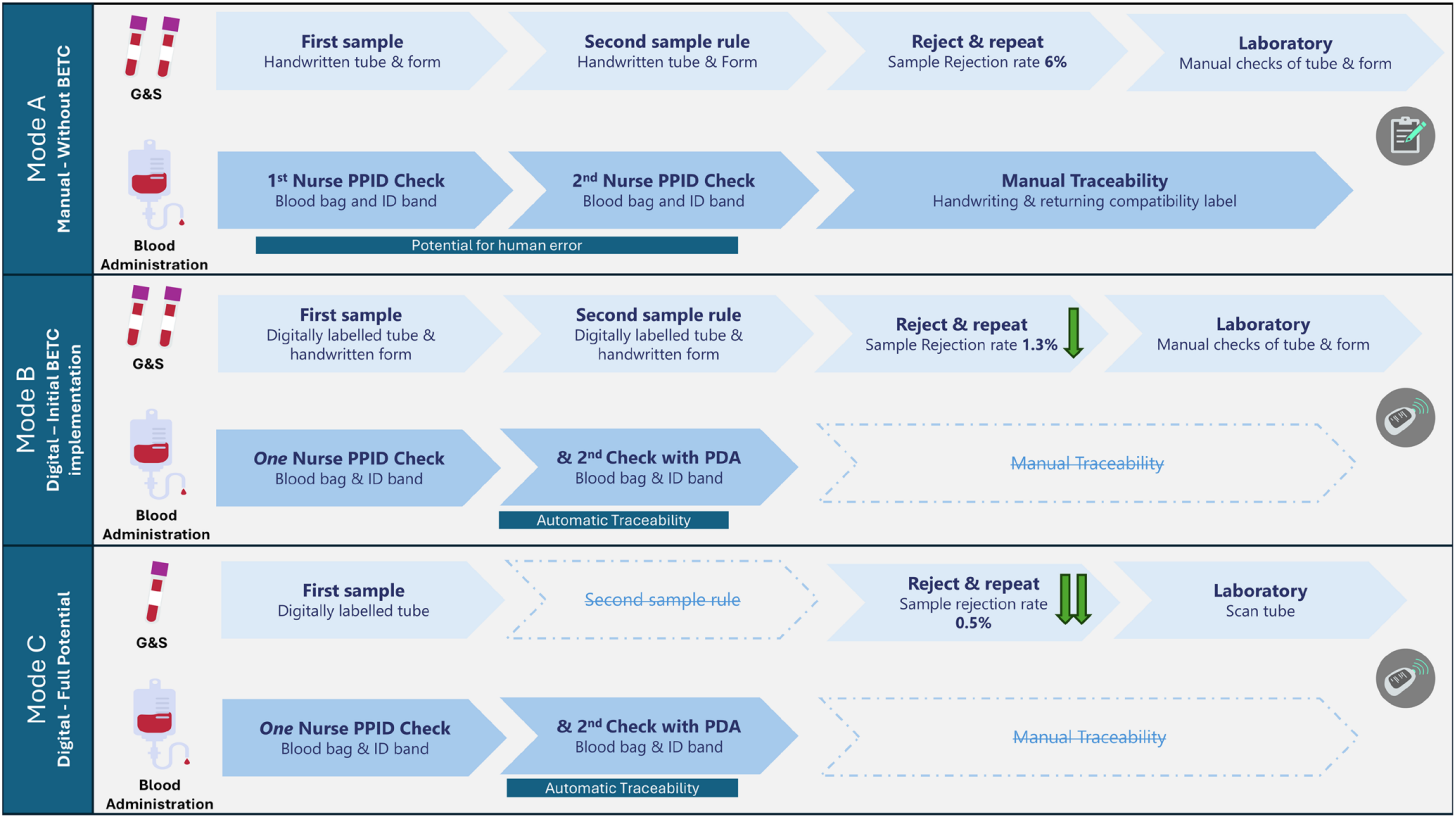
Overview of the transfusion pathway and the changes under implementation modes of BETC. BETC, bedside electronic transfusion check IT system; G&S, group and screen blood sample; ID, Identification; PPID, positive patient identification; PDA, personal digital assistant mobile device.

As part of implementing BETC across four London hospitals, which together form a single National Health Service (NHS) organisation (NHS trust), we mapped in detail transfusion workflow steps and quantified staff time savings associated with BETC use. Figure 1 illustrates the transfusion pathway under three levels of BETC support. Mode‐A represents the baseline without BETC: a manual, time‐consuming and error‐prone process, with associated risks including G&S sample rejection and incorrect blood transfusion. Mode‐B uses some BETC technological capability but retains some manual elements. This enables adoption in an initial proportion of clinical areas (coverage) and by a proportion of their staff (uptake). Mode‐C uses the full potential of BETC technology with scanning devices.

The main research question was how much staff time can be saved by implementing BETC under: (i) an initial implementation (Mode‐B), and (ii) planned fuller implementation (Mode‐C), compared with baseline manual processes (Mode‐A)? Staff practices observed during our data collection are also reported.

## METHODS

### Study design and setting

We adopted a prospective before‐after design,^6^ with three replications across sites. Haemonetics® BloodTrack® BETC was implemented between May 2022 and April 2025, with PDAs and mobile label printers. Following pre‐pilot and pilot phases, staff were trained sequentially on how to use the system across the four hospitals, starting with Hospital 1 (H1) in July–November 2023 and finishing with H4 (November 2024–April 2025). Research Ethics Committee approval (REC Reference Nr 22/NW/0138) and Health Research Authority approval (Nr 311 676) were obtained. Study protocol details were reported previously, plus practical lessons from the experience.^7^, ^8^, ^9^

### Process mapping

The transfusion pathway was observed, mapped and timed pre‐ and post‐implementation, using the Serious Hazards of Transfusion (SHOT) 10‐step transfusion pathway framework^10^ and established operations management methods,^11^, ^12^, ^13^, ^14^, ^15^ focusing on resource use (staff activity and timings) rather than sample turnaround times. High‐level process mapping was used previously in developing EBMS system specifications^16^ and for analysing prehospital blood supply for emergency‐response vehicles.^17^ One researcher used a continuous time–motion method^18^ to record the durations of tasks undertaken by individual members of staff.

A total of 40 G&S scenarios and 40 non‐emergency transfusions scenarios were observed at baseline (Mode‐A) in haematology day units (HDUs), split equally across hospitals H1–H4 (10 in each). Following initial implementation, 30 G&S scenarios and 30 non‐emergency transfusions were observed at H1–H3 (10 in each HDU). Emergency transfusions occur unpredictably; so, only 11 transfusion scenarios were observed in theatres (H1 and H3) for Mode‐A (baseline) and 10 with BETC Mode‐B (H1 and H3). Assistant Transfusion Practitioners recorded their work for follow‐up traceability over 16 weeks in autumn 2023, covering over 3000 blood‐component‐bag tags. Overall, the observational dataset captured over 3600 substep time measurements plus over 800 qualitative observations of practices. H4 had not reached the post‐initial‐implementation stage by the end of the time‐motion study so Mode‐B data were not available. However, it was included in the Mode‐A (baseline) dataset to examine potential differences and to enable modelling of projected Mode‐B and C impacts for the whole Trust. Subsequent PDA data suggest that BETC has had similar impacts at H4.

For each substep, we recorded staff role, time taken, consumables and any disruptions and protocol non‐compliance. Since Mode‐C will remove some staff tasks (rather than replacing or shortening tasks) no further round of time–motion data collection is necessary to assess the impact of this future, fuller use of BETC. Each substep was classified according to lean‐thinking principles^19^, ^20^, ^21^, ^22^ as a value step (required as part of the task), necessary waste (required by policy or in the absence of BETC support) or pure waste (repeating or fixing a task that was performed incorrectly). We applied a green/orange/red shading convention for these categories on our process map diagrams, as used in other clinical science pathway evaluations.^23^, ^24^, ^25^, ^26^

### Data collection

Transfusion activity data (G&S volumes, rejection rates and transfused units) were collected for a 12‐month baseline period (May 2022–April 2023) and 25 months post‐implementation (to July 2025) across the four hospitals. These longitudinal datasets were extracted from the Clinsys™ WinPath laboratory information management system (LIMS). We also had access to BETC data through the BloodTrack database of PDA activity; however, at the time of this initial implementation (Mode‐B), this database had not yet been integrated with the LIMS, precluding data cross‐referencing at G&S‐sample and blood‐unit level.

### Data analysis and modelling

The empirical data described above were collated and analysed with descriptive and inferential statistical methods for the staff timings data (Mann–Whitney and Kruskal–Wallis tests) and Statistical Process Control (SPC) analyses^22^, ^27^, ^28^ for the transfusion activity data. We cannot aggregate these data to estimate the impacts at the whole‐pathway and whole‐trust levels by summing task staff times—it requires modelling. This is because: patients could not be followed across pathway steps; there is not a one‐to‐one relationship between the G&S and blood administration (more G&S samples were taken than units transfused: 1.88 samples per blood component unit at baseline); and we wanted to project the eventual impacts of Mode‐B and Mode‐C for the whole Trust. For this last task, since H1 completed implementation first, its reduced G&S mislabelling rates and staff uptake were used to represent Mode‐B at the other hospitals. Although performance was not yet at this level across these other, subsequent data suggest they are all on a similar trajectory to reach similar values.

We used two modelling approaches: spreadsheet models to estimate aggregate mean time savings and Monte Carlo simulations^29^ to examine the variation around these means as we have in other studies.^30^ Pathway‐level spreadsheet models were first built (Appendix S1). These were then extended to organisation‐level models to estimate annual staff time savings under real‐world BETC use. In addition to the three BETC‐use modes (A: no BETC; B: initial; C: full), the model incorporated changes in coverage of BETC (the proportion of organisational activity designated for BETC implementation, since some clinical areas, such as outpatients, remained manual), and in staff uptake (defined as actual BETC use within covered areas, since use was not mandatory). This model combined the G&S sample and blood unit‐level time savings with the changes in mislabelling rejection rates, alongside the coverage and uptake data. Coverage data were provided by senior transfusion scientists.

The Monte Carlo simulation allowed generation of a very large number of possible combinations of individual empirical staff‐time‐used data points across steps and along the various possible pathway sections (e.g. a G&S sample that is rejected and re‐requested by the lab) using random sampling with replacement. Our results are based on 100 000 runs for each section.

Results are reported per G&S sample taken, per blood component unit transfused, across the entire transfusion pathway per unit transfused, and as aggregated annual savings in staff time and in G&S samples required across the four hospitals. For data analysis, modelling and presentation, we used Excel, PowerPoint, Lucidchart, R and STATA software.

## RESULTS

We set out to estimate how much staff time can be saved by BETC due to the reduced burden of safety checks and process data administration. We consider three states along the timeline of the phased implementation: baseline without BETC (Mode‐A use); initial (our Trust current state) implementation (Mode‐B use); and planned fuller implementation (Mode‐C use).

**FIGURE 2:**
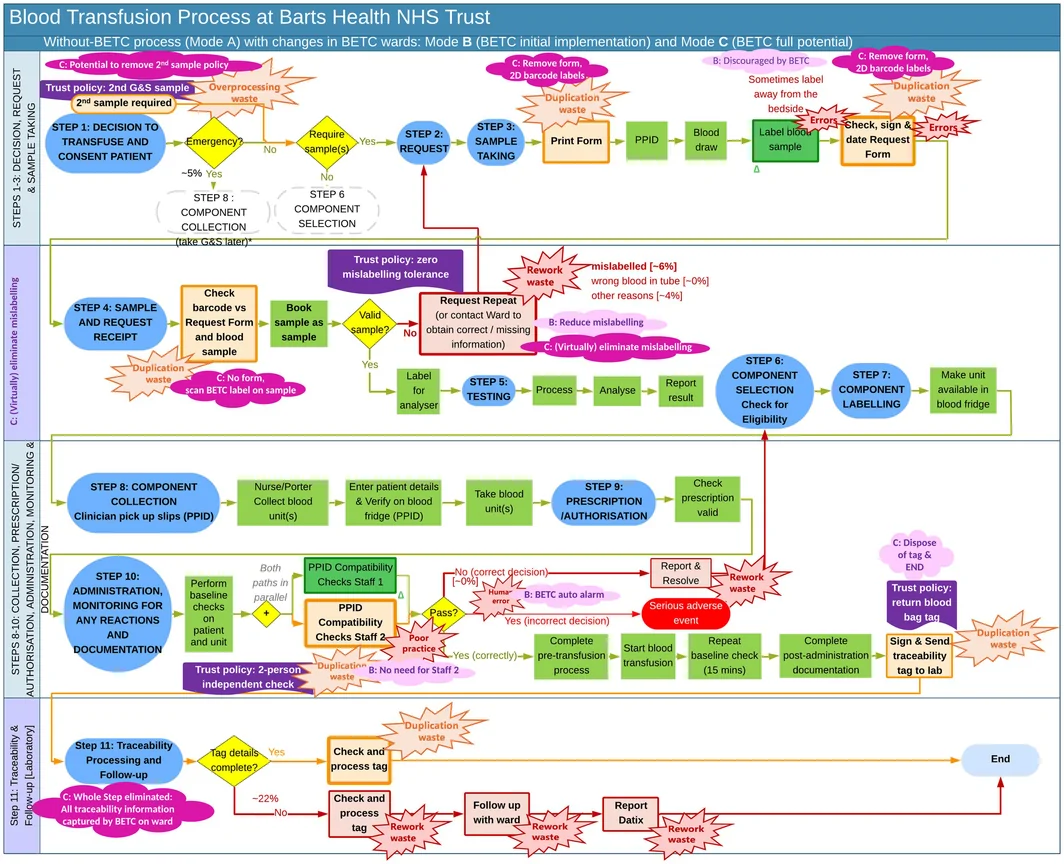
High‐level process map of the transfusion pathway. The purpose of the map is to highlight staff activity rather than end‐to‐end process durations (e.g. turnaround times). Blue ovals show the start of steps; their text in capitals are the 10 SHOT step titles. Green substeps show necessary (value‐adding) activities; a ∆ (delta) signifies a potential change (a reduction) in staff time used with BETC. Orange substeps and arrows show previously necessary but non‐value‐adding (‘waste’) activities and routes like duplication, removable with BETC. Red substeps and arrows show non‐value‐adding (‘waste’) activities and routes to fix errors (i.e., rework), removable or reduced with BETC. Deep purple shapes indicate Trust policies. Starbursts show issues with the pre‐BETC process and the clouds show the changes to the pre‐BETC process enabled by BETC to address these issues: Pale pink clouds are changes with initial BETC implementation (Mode‐B use) and brighter pink clouds are additional changes under full use (Mode‐C). *The process shows the non‐emergency transfusion pathway. For patients transfused under emergency conditions, after Step 1 the pathway jumps to an accelerated process for Steps 6–11, and the G&S sampling (if required, Steps 2–5) is carried out later in a similar manner to the non‐emergency pathway shown. BETC, bedside electronic transfusion check IT system; SHOT, Serious Hazards of Transfusion.

Figure 2 shows the high‐level transfusion process map, adopted from the SHOT 10‐Step framework^10^ with an 11th step (Step 11) for traceability and follow‐up added.^31^ Results show that BETC progressively streamlined clinical and laboratory processes, reducing waste under Mode‐B and further under Mode‐C (see Figures [Link], [Link] for detailed process maps for each step and mode). In Mode‐B, for the G&S sample‐taking step, manual labelling is replaced by the 2D barcoded labels (although accompanying paper forms were retained), while for blood administration the second‐nurse checks were replaced by PDA verification (see Table S1 for an overview of the changes across the modes). In Mode‐C, the G&S paper forms and second‐sample rule could also be eliminated.

There are two main parts to the empirical data gathered, which are combined to produce our aggregate staff time savings results. First, the staff times taken for substeps of the 11‐step process under the three modes of BETC use. The empirical data for the substeps (or groups of substeps) where we expected and observed changes in times, as indicated on Figures [Link], [Link], are shown in Figures S5 and S6. Considerable variation in staff time used within and between hospitals was observed, reflecting randomness of events, patient condition, staff experience and ward layout. BETC had a significant and material impact on staff time required for G&S and blood administration for all expected substeps. The data show reasonably similar times between sites (H1 to H4) and similar reductions from Mode‐A (_A suffix) to Mode‐B (_B). The limited formal‐study time window precluded Mode‐B data for the final implementation site (H4). The arrows show pooled reductions in times. For some substeps moving to Mode‐B or C means step removed, so to zero. The only non‐reduction is Staff 1 blood administration checks under emergency conditions (Figure S6, middle panel, top graph).

The other element is volume of work required (G&S samples, blood, traceability‐tag follow‐up). Our earlier paper showed H2‐H4 rejection rates following on with similar trajectories to H1,^8^ so we took H1 as representative. The volume‐weighted trust‐aggregate mislabelling rejection rate was 6.0% pre‐BETC (Mode‐A) and H1 subsequently achieved 1.3% as Mode‐B matured. For future Mode‐C performance, we took 0.5% conservatively; another NHS trust has reported 0.1%.^32^ Traceability follow‐up (22% under Mode‐A) was eliminated under BETC since PDAs automated data capture.

These data on faster and eliminated steps, together with uptake and reduced rejection rates, form the basis for modelling overall time‐savings. Table 1 summarises parameters used to model the aggregate annual time‐savings across the whole organisation (the four hospitals) with BETC.

**TABLE 1:** Parameters for the aggregate organisation‐level model of impacts of modes of BETC use.

Parameter	Mode‐A	Mode‐B	Mode‐C
G&S sample mislabelling rejection rates from BETC‐designated clinical areas	6.0%	1.3%	0.5%
Proportion of four hospitals' G&S activity covered by BETC‐designated clinical areas	0%	84.3%	94.7%
Proportion of hospitals' Blood Administration activity covered by BETC (as above)	0%	100%	100%
Actual uptake (usage) of BETC for G&S in BETC‐designated clinical areas	0%	85.2%	100%
Actual uptake (usage) of BETC for Blood Administration (as above)	0%	98.3%	100%
Proportion of valid G&S sample workload that is due to 2nd G&S Sample Policy	21.8%	21.8%	0%
Proportion of blood components administered requiring traceability follow‐up	22.2%	0%	0%

Abbreviation: BETC, bedside electronic transfusion check IT system; G&S, group and screen.

**FIGURE 3:**
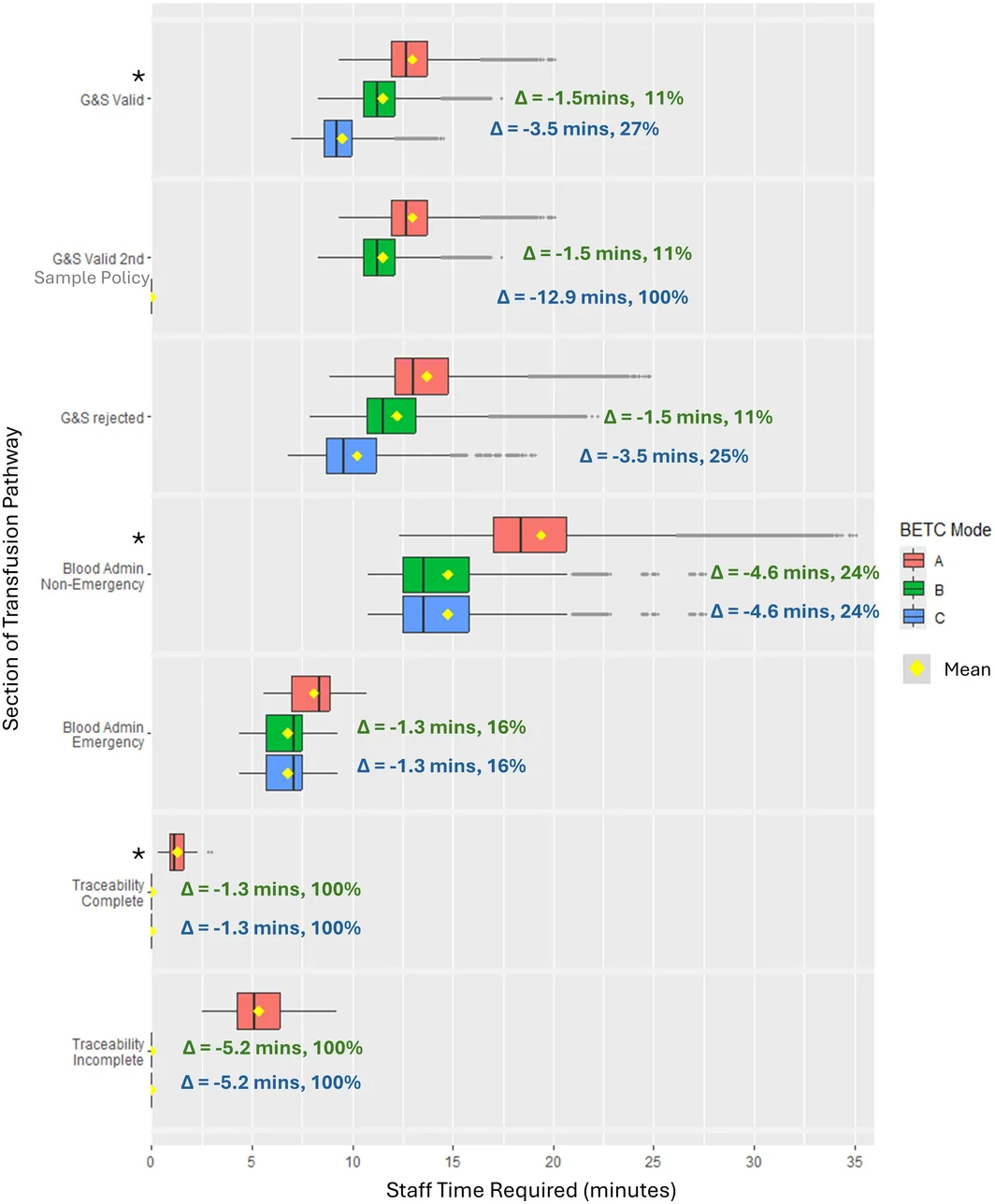
Staff time used in group & screen, blood administration and traceability follow‐up for sections and outcomes of the transfusion pathway. The box‐and‐whisker plots each represent 100 000 runs (possible combinations of the empirical step or substeps staff‐time datapoints), with ‘outliers’ beyond the whiskers, indicated by dots. A colour‐filled box represents the central 50% of the data, with the median indicated by a thicker vertical black bar. ∆ = Time savings per sample or blood unit relative to Mode‐A, using the means % = Time savings per sample or blood unit as proportion of time used under Mode‐A *Proportions are: G&S: In the Baseline (Mode‐A): 71% Valid (accepted by the lab); 20% Valid 2nd sample (accepted and required by 2‐sample policy); 6% Rejected by the lab due to mislabelling; 4% Rejected for other Reasons Blood Administration (all Modes): 95% transfused under Non‐Emergency conditions (regular pathway shown in Figure 2); 5% Emergency conditions (accelerated pathway) Traceability Follow‐up (Mode‐A only): 78% of blood‐bag tags returned to the lab have Complete information; 22% Incomplete (so require follow‐up work).

These modelled results are shown in Figure 3 (the Monte Carlo aggregation of empirical data to give estimates of variation) and Tables [Link], [Link] and Appendix S1 (spreadsheet modelling based on means). For G&S labelling, BETC reduced staff time by 1.5 min per sample (Mode‐B) through faster labelling checks, and 3.5 min under Mode‐C due to removal of the paper request form, corresponding to 11% and 25% time savings, respectively, for valid G&S samples and 11% and 27% for rejected samples versus baseline.

For blood administration, BETC reduced staff time by 4.6 min per non‐emergency unit (95% of units transfused) and 1.3 min per emergency unit (under Mode‐B and ‐C) through faster checks, removal of second‐staff verification and elimination of the need to return blood‐bag tags to the laboratory. For traceability, BETC saved 1.3 min per completed tag (78% of tags under Mode‐A) and 5.2 min per incomplete‐tag data‐remediation workflow. Overall, this is 4.5 min of staff time saved during transfusion and 2.1 min during traceability, representing 24% and 100% of baseline (Mode‐A) times respectively, with total savings of 6.6 min (Mode‐B and ‐C) per unit transfused (32% of Mode‐A).

Across the whole pathway, staff time saved per blood unit was 9.3 min (20% reduction) under Mode‐B and 11.4 min (25%) under C compared with baseline, excluding G&S sample reductions (Appendix S1). Including these, savings increased (prior to rounding) to 9.3 min (20%) under Mode‐B and 17.3 min (38%) under full implementation (Mode‐C) (Table 2).

**TABLE 2:** Trust‐level staff time savings from modes of BETC use.

Source task	Type of task	Type of saving				Uptake		Time saving per sample or unit				
			Total No. if 100% BETC coverage	Estimated BETC coverage				See Table S2	Total time saving
			Baseline staff time	45.42 mins Appendix S1	
See Appendix S1	See Table 1	Total No. with this BETC coverage	See Table 1	Total No. with this BETC uptake	mins	hrs	% of total time saving	% of baseline	
Implementation of BETC in Mode B: around 70% of G&S uses BETC, but not in full‐feature usage mode												
G&S Required (valid 1st samples)	Value	Less staff time to do the same job	73 762	84.3%	62 181	85.2%	52 979	1.48	**1307**	14. 6%		
Additional tests (valid)	Value	Less staff time to do the same job	2991	84.3%	2521	85.2%	2148	1.48	**53**	0.6%		
(Valid) 2nd samples required	(Currently) necessary duplication waste	Less staff time to do the same job	21 388	84.3%	18 030	85.2%	15 362	1.48	**379**	4.2%		
Rejections still occurring—mislabelling (despite BETC)	Rework waste	Less staff time to do the same job	1340	84.3%	1130	85.2%	962	1.48	**24**	0.3%		
Rejections occurring—other reasons	Rework waste	Less staff time to do the same job	3616	84.3%	3048	85.2%	2597	1.48	**64**	0.7%		
Rejections no longer occurring—mislabelling	Rework waste	Tasks avoided	5131	84.3%	4325	85.2%	3685	13.70	**841**	9.4%		
Rejections no longer occurring—others	Rework waste	Tasks avoided	186	84.3%	157	85.2%	134	13.70	**31**	0.3%		
Total G&S in baseline			108 414		91 393							
Transfusions Required	Value	Less staff time to do the same job	57 732	100.0%	57 732	98.3%	56 751	4.47	**4228**	47.2%		
Traceability no longer required	Necessary/duplication waste	Tasks avoided	57 732	100.0%	57 732	98.3%	56 751	2.14	**2023**	22.6%		
Total staff time released from direct transfusion activities									**8950**	hours		
Total staff time saved per unit transfused by the Trust									**9.30**	mins	**20%**	
G&S samples avoided							**3819**				**4%**	
Implementation of BETC in Mode C: around 95% of G&S uses BETC, in full‐feature usage mode												
G&S Required (valid 1st samples)	Value	Less staff time to do the same job	73 762	94.7%	69 853	100%	69 853	3.47	**4040**	24.3%		
Additional tests (valid)	Value	Less staff time to do the same job	2991	94.7%	2832	100%	2832	3.47	**164**	1.0%		
(Valid) 2nd samples now unnecessary	(Formerly) necessary duplication waste	Tasks avoided	21 388	94.7%	20 254	100%	20 254	13.00	**4390**	26.4%		
Rejections still occurring—mislabelling (despite BETC)	Rework waste	Less staff time to do the same job	400	94.7%	379	100%	379	3.47	**22**	0.1%		
Rejections occurring—other reasons	Rework waste	Less staff time to do the same job	2804	94.7%	2655	100%	2655	3.47	**154**	0.9%		
Rejections no longer occurring—mislabelling	Rework waste	Tasks avoided	6071	94.7%	5749	100%	5749	13.70	**1313**	7.9%		
Rejections no longer occurring—others	Rework waste	Tasks avoided	998	94.7%	945	100%	945	13.70	**216**	1.3%		
Total G&S in baseline			108 414		102 668							
Transfusions Required	Value	Less staff time to do the same job	57 732	100%	57 732	100%	57 732	4.47	**4301**	25.8%		
Traceability no longer required	(Formerly) necessary/duplication waste	Tasks avoided	57 732	100%	57 732	100%	57 732	2.14	**2058**	12. 4%		
Total staff time released from direct transfusion activities									**16 657**	hrs		
Total staff time saved per unit transfused by the Trust									**17.31**	mins	**38%**	
G&S samples avoided							**26 949**				**25%**	

*Note*: The most important results are indicated in bold. Green/orange/red shading emphasises value work / (currently) necessary waste / pure waste.

Results (Table 2) show these aggregate annual time‐savings estimates are 8950 h with Mode‐B (20% reduction) and 16 650 h with Mode‐C (38%), versus Mode‐A. Much of this comes from reduced G&S samples (3800 fewer under Mode‐B; 26 950 Mode‐C) due to reduction in rejection rates (and so fewer repeat samples required) and removal of the second‐sample policy, representing 4% and 25% fewer samples respectively.

### Observations of practice

Under Mode‐A, transfusion compliance with manual safety protocols for PPID was poor. During G&S, 43% of patients (17/40) did not have wristbands on and 5% of samples (2/40) were labelled away from the patient's bedside. During blood administration, no second staff checks (0/40) were considered truly independent, with additional deviations including delayed documentation and task interruption. These lapses could cause errors, posing safety risks. BETC removed the need for independent checks, thus reducing task burden, improving safety and eliminating serious issues observed.

## DISCUSSION

This study aimed to examine the transfusion pathway across four hospitals in one of the largest NHS organisations (NHS trusts) in the United Kingdom, quantifying the impact of BETC systems on staff‐time saved, critical evidence for assessing value for money. To our knowledge, this is the first such detailed exploration.

In this study, we show that the benefits of BETC depend heavily on its use (coverage of activity in the hospital and uptake by clinical staff). Under initial (current) implementation, staff time per blood unit was reduced by 9.3 min across the whole transfusion pathway. At the aggregate organisation level (all four hospitals), this corresponds to annual savings of 8950 h, with 3800 fewer G&S samples, resulting in a 4% reduction in required G&S repeats. Under full uptake, we predict staff‐time savings increase to 17.3 min across the whole pathway (per unit), with annual organisation‐level time‐savings being 16 650 h for the four hospitals, driven largely by the removal of the second G&S sample policy, resulting in a 25% reduction in G&S workload.

In this study, we saw that G&S labelling uptake was slower than expected, limiting both time‐savings and rejection‐rate reductions (see also^8^). Mandating use following training may be required to enhance uptake and benefits. In contrast, uptake of BETC for blood administration was faster, reflecting strong practical and safety incentives for staff. BETC also virtually eliminated manual traceability follow‐up.^33^, ^34^ Finally, LIMS rejections data highlight hospital locations with BETC adoption and use issues, underscoring the potential value of targeted quality‐improvement and learning‐and‐support cycles reported elsewhere.^32^, ^34^

Studies describing BETC resource analyses are limited. A Hong Kong study (1997–1999) found no impact of barcode systems on G&S time, though, unlike our study, this relied on self‐reported start‐to‐finish times rather than direct observation of staff activity times,^35^ making comparison with our results difficult.

Health Technology Wales^3^ drew on previous work in English hospitals,^32^, ^33^ reporting full‐implementation results (cf. Mode‐C) of 50% staff time reduction for blood administration PPID checks, very similar to our results. They also reported large overall reductions in staff time (clinical: 58 to 17 min; laboratory: 59 to 17 min) ‘per transfusion’, broadly aligning with our whole‐pathway analysis (46 to 34 min). However, their changes also included electronic remote blood fridges, so the results are not directly comparable.

Uptake also differed. The Hong Kong study reported 90% BETC uptake, limited mainly by hardware issues (e.g. barcode scanner batteries). By the same point in time, 20 months post‐implementation (Mode‐B), our first hospital (H1) had lower uptake for G&S than Oxford (70% vs. their 95%), but slightly higher for blood administration (98% vs .95%).^33^

Poor compliance with manual transfusion protocols is common across the NHS.^36^ The Hong Kong and Oxford studies both observed dramatic improvements in compliance with blood administration checks: Oxford reported 12% without BETC vs. 100% with BETC,^32^, ^34^ closely mirroring our findings (0% vs. 100%).

### Limitations

This study has some limitations. Although we collected detailed process mapping data, we were unable to do this for every G&S sample taken and blood unit administered in the four hospitals due to the very large volume of activities (around 110 000 G&S samples and around 60 000 units annually) and limited research resources. We considered using detailed substep PDA‐timestamp data that can be extracted from BloodTrack, but resource constraints precluded mapping and analysing these very large volumes of raw data; future researchers might explore this.

It is important to analyse work as it *is* done, rather than how it *should* be done, particularly in the NHS, where staff under pressure often adopt ‘workarounds’.^37^ However, as with all observational time‐motion studies, behaviour may have be influenced by awareness of being observed (the Hawthorne Effect).^38^ However, ward nurses were generally very occupied in the process and observed non‐compliance with safety protocols, particularly for blood administration, suggests little attempt to perform steps ‘by the book’ for the observer's benefit. Clinical activities are generally intermittent and unpredictable, particularly in emergency environments. We could not capture all the variety of possibilities, but the purpose of process mapping is to capture what matters most for study's purpose. While mapping the entire pathway was valuable, in retrospect perhaps focusing on those substeps affected by BETC, rather than the whole pathway, may have been more efficient. Either way, this study provides valuable information that can be used to quantify the health economic benefits of implementing BETC in hospitals.

## CONCLUSIONS

Our study provides the first large‐scale, detailed quantification of the impact of BETC on the transfusion pathway, demonstrating benefits under actual implementation and its potential under full adoption. Our findings of similar impacts across phased implementations at four large hospital sites suggest that similar benefits are feasible elsewhere. These findings can support other organisations preparing cost–benefit evaluations, while highlighting the need for decisive, comprehensive implementation to maximise patient safety, staff efficiency and value for money.

## AUTHOR CONTRIBUTIONS

**Florence Oyekan:** Data curation; investigation; validation; writing – review and editing. **Louise Bowles:** Conceptualization; validation; writing – review and editing. **Michael F. Murphy:** Validation; writing – review and editing. **Helinor McAleese:** Validation. **Laura Green:** Conceptualization; methodology; investigation; validation; supervision; funding acquisition; project administration; resources; writing – review and editing. **Josephine McCullagh:** Conceptualization; investigation; formal analysis; project administration; writing – review and editing. **Simon J. Stanworth:** Validation; writing – review and editing. **Montasir Ahmed:** Investigation; formal analysis; writing – review and editing. **Yan Feng:** Supervision; writing – review and editing. **Nathan Proudlove:** Conceptualization; methodology; software; data curation; validation; formal analysis; supervision; visualization; writing – original draft.

## FUNDING INFORMATION

Funding was provided by Barts Charity and NHS Charities Together (through Barts Charity).

## CONFLICT OF INTEREST STATEMENT

Mike Murphy has received consultancy fees from Haemonetics for speaking at webinars and other events. All other authors declare no conflict of interest.

## Supporting information


**Appendix S1.** Whole pathway level impact of BETC.

**Figure S1.** Detailed process map of Steps 1–3—Clinical area G&S sample taking.

**Figure S2.** Detailed process map of Steps 4 & 5—Laboratory processing and testing of G&S samples.

**Figure S3.** Detailed process map of Step 10—Clinical area blood administration to patient.

**Figure S4.** Detailed process map of Step 11—Laboratory traceability documentation and follow‐up.

**Figure S5.** Steps 3–5—G&S sampling and testing substep duration data.

**Figure S6.** Steps 10 & 11—Blood administration and traceability substep duration data.

**Table S1.** Changes to transfusion pathway following implementation of bedside electronic transfusion checks (BETC), considering the three modes and extents of use.

**Table S2.** Overview of step durations under each BETC mode and time savings when moving from one mode to another.

**Table S3.** Impacts of BETC implementation on the pathway on Steps 3–5—Clinical area G&S sample taking and Laboratory processing and Testing, split by valid versus rejected.

**Table S4.** Impacts of BETC implementation on the pathway on Step 10—Clinical area blood administration to patient, split by non‐emergency versus emergency transfusion.

**Table S5.** Impacts of BETC implementation on the pathway on Step 11—Clinical area blood administration to patient—Laboratory traceability documentation and follow‐up, split complete vs incomplete tag information.

## Data Availability

The data that support the findings of this study are available from the corresponding author upon reasonable request.
