# Supplementary material for: Efficiency and safety gains from bedside electronic transfusion checks in four hospitals in England
Source: Br J Haematol. 2026 Aug 6;209(3):1311–20. doi: 10.1111/bjh.70681 (PMC13570133; doi:10.1111/bjh.70681)
Supplement: Supplementary file 1 [file BJH-209-1311-s002.docx]

**Table S5:	Impacts of BETC implementation on the pathway on Step 11 - Clinical-area blood administration to patient**

– Laboratory traceability documentation and follow-up, split by Complete vs Incomplete Tag Information

| **Traceability (Step 11)** | | *removed* |  |  | **Total (mins)** | **Total (mins)** |
| --- | --- | --- | --- | --- | --- | --- |
|  |  | *removed* |  |  |  | **All sub-steps** |
| *Step codes* | **Tag Info Complete** | ***11.01*** |  |  |  |  |
| Description |  | **Check and Process Tag** |  |  |  |  |
| *Step codes* | **Tag Info Incomplete** | ***11.02*** | ***11.03*** | ***11.04*** |  |  |
| Description |  | **Check and Process Tag** | **Follow-up with ward** | **Datix (when necessary)** |  |  |
| **STAFF TIME (mins)** | |  |  |  |  |  |
| **Mode A** | **Tag Info Complete** | 1.29 |  |  | 1.29 |  |
|  | **Tag Info Incomplete** | 3.03 | 1.69 | 0.43 | 5.15 |  |
| **Mode B** | **Tag Info Complete** | 0 |  |  | 0 |  |
|  | **Tag Info Incomplete** | 0 | 0 | 0 | 0 |  |
| **Mode C** | **Tag Info Complete** | 0 |  |  | 0 |  |
|  | **Tag Info Incomplete** | 0 | 0 | 0 | 0 |  |
| **TIME SAVINGS (mins)** | |  |  |  |  | % of Mode A |
| **A -> B** | **Tag Info Complete** | 1.29 |  |  | **1.29** | 100% |
|  | **Tag Info Incomplete** | 3.03 | 1.69 | 0.43 | **5.15** | 100% |
| **B -> C** | **Tag Info Complete** | 0 |  |  | 0 | 0% |
|  | **Tag Info Incomplete** | 0 | 0 | 0 | 0 | 0% |
| **A -> C** | **Tag Info Complete** | 1.29 |  |  | 1.29 | 100% |
|  | **Tag Info Incomplete** | 3.03 | 1.69 | 0.43 | 5.15 | 100% |
|  |  |  |  |  |  |  |
| Orange: necessary waste (removed with BETC) | | |  |  |  |  |
| Dark red: re-work waste (removed with BETC) | | |  |  |  |  |
| No decimal places means zero due to sub-step removal or no change | | | | |  |  |
| Decimal places means estimated from empirical data | | | |  |  |  |
