# Supplementary material for: Efficiency and safety gains from bedside electronic transfusion checks in four hospitals in England
Source: Br J Haematol. 2026 Aug 6;209(3):1311–20. doi: 10.1111/bjh.70681 (PMC13570133; doi:10.1111/bjh.70681)
Supplement: Supplementary file 2 [file BJH-209-1311-s005.docx]

**Table S4:	Impacts of BETC implementation on the pathway on Step 10**

- Clinical-area blood administration to patient, split by Non-emergency vs emergency transfusion

| **Blood Administration (Step 10)** | | *continuing* | *faster?* | *removed* | *removed* | **Total (mins)** | **Total (mins)** |
| --- | --- | --- | --- | --- | --- | --- | --- |
|  |  |  |  |  |  | **Direct** | **All sub-steps** |
| *Step codes* | **Non-emergency** | *10….* | ***10.91*** | ***10.92*** | ***10.25*** |  |  |
| Description |  | Rest of step | **Staff 1 Checks** | **Staff 2 (Independent Check)** | **Traceability on Ward** |  |  |
| *(constituent sub-steps)* | | = 10.02 +10.03 +10.04 +10.05 +10.09 +10.23 +10.24 +10.26 +10.27 +10.28 +10.29 | = 10.18 (+10.18b) +10.19 +10.20 +10.21 +10.22 | = 10.19 +10.20 +10.21 +10.22 |  |  |  |
| *Step codes* | **Emergency** | *10….* | ***10.91*** | ***10.92*** | ***10.25*** |  |  |
| Description |  | Rest of step | **Staff 1 Checks** | **Staff 2 (Independent Check)** | **Traceability on Ward** |  |  |
| *(constituent sub-steps)* | | = 10.03 +10.23 +10.27 +10.29 | = (10.05) +10.06 +10.18 (+10.18b) +10.19 +10.20 +10.21 +10.22 | = 10.19 +10.20 +10.21 +10.22 |  |  |  |
| **STAFF TIME (mins)** | |  |  |  |  |  |  |
| **Mode A** | **Non-emergency** | 13.15 | 3.30 | 2.64 | 0.31 | 6.25 | 19.40 |
|  | **Emergency** | 5.82 | 0.96 | 0.90 | 0.39 | 2.25 | 8.07 |
| **Mode B** | **Non-emergency** | 13.15 | 1.61 | 0 | 0 | 1.61 | 14.76 |
|  | **Emergency** | 5.82 | 0.96 | *0* | 0 | 0.96 | 6.78 |
| **Mode C** | **Non-emergency** | 13.15 | 1.61 | 0 | 0 | 1.61 | 14.76 |
|  | **Emergency** | 5.82 | 0.96 | 0 | 0 | 0.96 | 6.78 |
| **TIME SAVINGS (mins)** | |  |  |  |  |  | **% of Mode A** |
| **A -> B** | **Non-emergency** |  | 1.69 | 2.64 | 0.31 | 4.64 | 24% |
|  | **Emergency** |  | 0.00 | 0.90 | 0.39 | 1.29 | 16% |
| **B -> C** | **Non-emergency** |  | 0 | 0 | 0 | 0 | 0% |
|  | **Emergency** |  | 0 | 0 | 0 | 0 | 0% |
| **A -> C** | **Non-emergency** |  | 1.69 | 2.64 | 0.31 | 4.64 | 24% |
|  | **Emergency** |  | 0.00 | 0.90 | 0.39 | 1.29 | 16% |
