# Supplementary material for: Efficiency and safety gains from bedside electronic transfusion checks in four hospitals in England
Source: Br J Haematol. 2026 Aug 6;209(3):1311–20. doi: 10.1111/bjh.70681 (PMC13570133; doi:10.1111/bjh.70681)
Supplement: Supplementary file 3 [file BJH-209-1311-s008.docx]

**Table S2:** Overview of step durations under each BETC mode and time savings when moving from one mode to another

|  |  | **Baseline Proportions** | **Durations (mins)** | | | **Time savings between**  **Modes (mins)** | | | **% time saving**  **relative to Mode-A** | | |
| --- | --- | --- | --- | --- | --- | --- | --- | --- | --- | --- | --- |
| **Steps** |  |  | Mode-A | Mode-B | Mode-C | A -> B | B -> C | A -> C | A -> B | B -> C | A -> C |
| **3-5**  **G&S**  **and**  **Testing** | Valid 1st G&S / additional test samples | *71%* | 12.9 | 11.5 | 9.5 | 1.5 | 2.0 | 3.5 | 11% | 15% | 27% |
|  | Valid 2nd G&S (G&S policy) | *20%* | 12.9 | 11.5 | n/a | 1.5 | 11.5 | 12.9 | 11% | 89% | 100% |
|  | Rejected & Repeated G&S (mislabelled) | *6%* | 13.7 | 12.2 | 10.2 | 1.5 | 2.0 | 3.5 | 11% | 15% | 25% |
|  | Rejected & Repeated G&S (other reason) | *4%* | 13.7 | 12.2 | 10.2 | 1.5 | 2.0 | 3.5 | 11% | 15% | 25% |
|  | G&S: Aggregate |  | 13.0 |  |  |  |  |  |  |  |  |
| **10**  **Blood**  **Administration** | Transfusion: Non-Emergency | *95%* | 19.4 | 14.8 | 14.8 | 4.6 | 0 | 4.6 | 24% | 0% | 24% |
|  | Transfusion: Emergency | *5%* | 8.1 | 6.8 | 6.8 | 1.3 | 0 | 1.3 | 16% | 0% | 16% |
|  | Transfusion: Aggregate |  | 18.8 | 14.4 | 14.4 | 4.5 | 0 | 4.5 | 24% | 0% | 24% |
| **11**  **Traceability**  **and follow-up** | Traceability: Tag Info Complete | *78%* | 1.3 | n/a | n/a | 1.3 | 0 | 1.3 | 100% | 0% | 100% |
|  | Traceability: Tag Info Incomplete | *22%* | 5.2 | n/a | n/a | 5.2 | 0 | 5.2 | 100% | 0% | 100% |
|  | Traceability: Aggregate |  | 2.1 |  |  | 2.1 | 0 | 2.1 | 100% | 0% | 100% |

Valid = G&S sample not rejected by lab.

Green: required work successfully completed. Modes-B & C: less time-consuming with BETC.

Orange: necessary work in G&S due to Policy. Mode-B: less time-consuming with BETC. Mode-C: potential for removal of this task.

Traceability requirements with manual process. Modes-B&C: task removed as traceability is captured by PDAs.

Light red: G&S sample that is rejected due to non-labelling-reason rejection. Modes-B & C: still occur, but less time-consuming with BETC.

Dark red: G&S sample that is rejected due to labelling-reason rejection. Modes-B & C: still can occur, but less time-consuming with BETC.

remedial traceability work to obtain information missing from tags. Modes-B&C: task removed as PDAs capture complete information.

All duration data are derived from the observed timings of sub-steps. “n/a” indicates 0 because this task has been removed (Mode-B) or is expected to be (Mode-C). Data were fairly similar across the 4 sites (see Figures S2 and S3), with no site standing out as materially different. Baseline proportions are from the laboratory information system (G&S), expert judgement (non-emergency vs emergency) or several months of aggregate records (traceability).

Overall times for G&S Modes-B & C not calculated as the proportions change from the baseline values. Similarly, total savings along the pathway are not summed here as the ratio of G&S sample to units of blood administered is not 1:1 and varies across the modes.
