# Supplementary material for: Efficiency and safety gains from bedside electronic transfusion checks in four hospitals in England
Source: Br J Haematol. 2026 Aug 6;209(3):1311–20. doi: 10.1111/bjh.70681 (PMC13570133; doi:10.1111/bjh.70681)
Supplement: Supplementary file 4 [file BJH-209-1311-s009.docx]

**Table S1.**	Changes to transfusion pathway following implementation of Bedside Electronic Transfusion Checks (BETC), considering the three modes and extents of use

| **Step and Sub-Step** | **Mode-A:**  **Without BETC** | **Mode-B:**  **With BETC – actual implementation** | **Mode-C:**  **With BETC - full potential** |
| --- | --- | --- | --- |
| ***BETC Coverage*** | *None* | *most G&S, almost all Blood Administration* | *almost all G&S, all Blood Administration* |
| **i) Direct Impact - on waste removal from value-work pathway (green arrow route on Fig 1)** | | | |
| **STEP 3 (Clinical area): SAMPLE TAKING AND LABELLING** | | | |
| Label tube(s) | Hand label tubes | **BETC 2D barcoded labels** | **(Already changed)** |
| Complete Paper Request Form | Fill in, accompanies sample tube | Continued | **Removed (2D labels sufficient)** |
| 2^nd^ Sample Rule | Required for some patients  Applied inappropriately | Continued | **Remove** |
| **STEP 4 (Laboratory): SAMPLE AND REQUEST RECEIPT** | | | |
| Cross-check and enter details | Scan Paper Request Form 1D barcode, check details vs tube label, enter details into LIMS | Continued | **Just scan 2D label barcode (no paper form)** |
| **STEP 10 (Clinical area): ADMINISTRATION, MONITORING FOR ANY REACTIONS AND DOCUMENTATION** | | | |
| Checks pre-transfusion | Require 2^nd^ nurse | **Removed (PDA instead of 2^nd^ nurse)** | **(Already changed)** |
| **Step 11 (Laboratory): Traceability and Follow-up** | | | |
| Monitor Traceability | Check blood bag tags | **Removed (PDA captures information in Step 10)** | **(Already changed)** |
| **ii) Indirect Impact - on error rework (waste) pathways (red arrow routes)** | | | |
| **STEP 3 (Clinical area): SAMPLE TAKING** | | | |
| Repeat of whole step | High rate of mislabelling [inc. form issues]: 6.0% | **Reduced rate of mislabelling, but also new types: 1.3%** | **Mislabelling almost eliminated (& form removed): 0.5%** |
| **STEP 4 (Laboratory): SAMPLE AND REQUEST RECEIPT** | | | |
| Request Repeat of G&S sampling (Step 3) | High rate of mislabelling [inc. form issues]: 6.0% | **Reduced rate of mislabelling, but also new types: 1.3%** | **Mislabelling almost eliminated (& form removed): 0.5%** |
| **STEP 10 (Clinical area): ADMINISTRATION, MONITORING FOR ANY REACTIONS AND DOCUMENTATION** | | | |
| Contact lab to reissue [and repeat of Steps 8, 9 & 10 to this point] | [very rare] | [even rarer] | [even rarer] |
| **Step 11 (Laboratory): Traceability and Follow-up** | | | |
| Follow-up incomplete information | Follow-up missing information: 22% | **Removed** | **(Already changed)** |

*G&S: group and screen; LIMS: laboratory information management system*
