# Supplementary material for: Efficiency and safety gains from bedside electronic transfusion checks in four hospitals in England
Source: Br J Haematol. 2026 Aug 6;209(3):1311–20. doi: 10.1111/bjh.70681 (PMC13570133; doi:10.1111/bjh.70681)
Supplement: Supplementary file 5 [file BJH-209-1311-s011.docx]

**Table S3:	Impacts of BETC implementation on the pathway on Steps 3-5**

Clinical-area G&S sample taking and Laboratory processing and Testing, split by Valid vs Rejected G&S sample

| **G&S (Steps 3-5)** | | *continuing* | *continuing* | *faster?* | *removed* | *removed* | *removed* | *continuing* | *continuing* | *continuing* | **Total (mins)** | **Total (mins)** |
| --- | --- | --- | --- | --- | --- | --- | --- | --- | --- | --- | --- | --- |
|  |  |  |  |  |  |  |  |  | *continuing* |  | **Direct** | **All sub-steps** |
| *Step codes* | **Valid G&S** | *3….* | *3.08* | ***3.91*** | ***3.16*** | ***3.90*** | ***4.02*** | *4.01* | *4….* | *5….* |  |  |
| Description |  | *Rest of step* | *PPID & Blood Draw* | **Scan & Label** | **Check Label** | **Process Form on Ward** | **Process Form in Lab** | Scan | Book, label | Testing |  |  |
| *(constituent sub-steps)* | |  |  | = 3.11 +3.12 |  | = 3.02 +3.03  +3.04 +3.17 |  |  | = 4.03  + 4.05 |  |  |  |
| *Step codes* | **Rejected & Repeated G&S** | *3….* | *3.08* | ***3.91*** | ***3.16*** | ***3.90*** | ***4.02*** | *4.01* | *4.04* |  |  |  |
| Description |  | *Rest of step* | *PPID & Blood Draw* | **Scan & Label** | **Check Label** | **Process Form on Ward** | **Process Form in Lab** | Scan | Book, reject, re-request |  |  |  |
| *(constituent sub-steps)* | |  |  | = 3.11 +3.12 |  | = 3.02 +3.03  +3.04 +3.17 |  |  |  |  |  |  |
| **STAFF TIME (mins)** | |  |  |  |  |  |  |  |  |  |  |  |
| **Mode A** | **Valid G&S** | 5.38 | 1.94 | 1.48 | 0.75 | 1.85 | 0.14 | 0.03 | 1.06 | 0.30 | 6.16 | 12.93 |
|  | **Rejected & Repeated G&S** | 5.38 | 1.94 | 1.48 | 0.75 | 1.85 | 0.14 | 0.03 | 2.13 |  | 6.16 | 13.70 |
| **Mode B** | **Valid G&S** | 5.38 | 1.94 | 0.75 | n/a | 1.85 | 0.14 | 0.03 | 1.06 | 0.30 | 4.68 | 11.45 |
|  | **Rejected & Repeated G&S** | 5.38 | 1.94 | 0.75 | n/a | 1.85 | 0.14 | 0.03 | 2.13 |  | 4.68 | 12.22 |
| **Mode C** | **Valid G&S** | 5.38 | 1.94 | 0.75 | n/a | n/a | n/a | 0.03 | 1.06 | 0.30 | 2.69 | 9.46 |
|  | **Rejected & Repeated G&S** | 5.38 | 1.94 | 0.75 | n/a | n/a | n/a | 0.03 | 2.13 |  | 2.69 | 10.23 |
| **TIME SAVINGS (mins)** | |  |  |  |  |  |  |  |  |  |  | **% of Mode A** |
| **A -> B** | **Valid G&S** |  |  | 0.73 | 0.75 | 0 | 0 |  |  |  | **1.48** | **11%** |
|  | **Rejected & Repeated G&S** |  |  | 0.73 | 0.75 | 0 | 0 |  |  |  | **1.48** | **11%** |
| **B -> C** | **Valid G&S** |  |  | 0.00 | 0 | 1.85 | 0.14 |  |  |  | 1.99 | 15% |
|  | **Rejected & Repeated G&S** |  |  | 0.00 | 0 | 1.85 | 0.14 |  |  |  | 1.99 | 15% |
| **A -> C** | **Valid G&S** |  |  | 0.73 | 0.75 | 1.85 | 0.14 |  |  |  | **3.47** | **27%** |
|  | **Rejected & Repeated G&S** |  |  | 0.73 | 0.75 | 1.85 | 0.14 |  |  |  | **3.47** | **25%** |
